# Grain Boundary Specific Segregation in Nanocrystalline Fe(Cr)

**DOI:** 10.1038/srep34642

**Published:** 2016-10-06

**Authors:** Xuyang Zhou, Xiao-xiang Yu, Tyler Kaub, Richard L. Martens, Gregory B. Thompson

**Affiliations:** 1 The University of Alabama, Department of Metallurgical & Materials Engineering, Tuscaloosa, AL USA; 2 The University of Alabama, Central Analytical Facility, Tuscaloosa, AL USA

## Abstract

A cross-correlative precession electron diffraction – atom probe tomography investigation of Cr segregation in a Fe(Cr) nanocrystalline alloy was undertaken. Solute segregation was found to be dependent on grain boundary type. The results of which were compared to a hybrid Molecular Dynamics and Monte Carlo simulation that predicted the segregation for special character, low angle, and high angle grain boundaries, as well as the angle of inclination of the grain boundary. It was found that the highest segregation concentration was for the high angle grain boundaries and is explained in terms of clustering driven by the onset of phase separation. For special character boundaries, the highest Gibbsain interfacial excess was predicted at the incoherent ∑3 followed by ∑9 and ∑11 boundaries with negligible segregation to the twin and ∑5 boundaries. In addition, the low angle grain boundaries predicted negligible segregation. All of these trends matched well with the experiment. This solute-boundary segregation dependency for the special character grain boundaries is explained in terms of excess volume and the energetic distribution of the solute in the boundary.

Grain boundary energy plays a significant role in controlling grain morphology and growth behavior in polycrystalline materials. This energy is directly linked to the grain boundary character between the polycrystalline grains. Since the relative grain size can be crucial in regulating the strength and electrical management of materials, particularly at small length scales^1^,^2^,^3^,^4^, the ability to manage grain boundary energy is essential. In recent years, the use of solute segregation as a means to alter the grain boundary energy to control the grain size and shape has been shown to help stabilize nanocrystalline grains against growth at high homologous temperatures^5^,^6^,^7^,^8^. Though these reports assume that solute segregation is isotropic and equivalent, in reality grain boundaries are diverse in energy, structure, and mobility^9^,^10^,^11^,^12^. This would imply that solute segregation would be a function of grain boundary type. However, quantifying the solute segregation to specific boundaries to determine this solute-orientation dependence is an arduous task because one must have both high chemical sensitivity and high spatial resolution to detect the solute in the known grain boundary. Of the available techniques, atom probe tomography (APT) is capable in providing the local grain boundary composition but only rarely is able to provide the necessary atomic spatial resolution to identify grain boundary misorientation^13^. In the vast majority of APT data sets published, the identification of different lattice planes from different grains is either not done or is not possible because of inherent reconstruction issues associated with the APT technique^14^. In contrast, electron diffraction and high resolution transmission electron imaging can provide the grain boundary misorientation and even solute identification^15^,^16^,^17^, but the two-dimensional projection limits the depth prospective of the solute over the grain boundary and even the determination of the inclination angle of the boundary. In addition, the imaging of the boundary also requires that it is orientated to a specific crystallographic zone axis to meet the imaging requirements. As with APT, electron-based imaging also has experimental limitations in its ability to quantify solute-boundary interactions.

To overcome those issues, in recent years, cross-correlated electron microscopy with atom probe has been used^18^,^19^. For example, Li *et al*.^18^ used electron backscatter diffraction (EBSD) to identify prior austenite grain boundaries in steel from which site specific focus ion beam (FIB) lift out samples were prepared into the APT required needle-shape geometries. The results revealed B and Mo segregation to these boundaries. More recently, Herbig *et al*.^19^ directly FIB lifted out and annular ion milled a ferrite steel APT specimen and directly performed transmission electron diffraction of the APT specimen needle which subsequently mapped out the grain boundary misorientations within the tip. The tip was field evaporated and the atom map was reconstructed with *a priori* knowledge of the grain boundary types revealing C segregation behavior between low and high angle grain boundaries.

In this paper we further those efforts by quantifying the solute segregation to specific nanocrystalline grains using precession electron diffraction (PED)^20^,^21^,^22^ and APT. Unlike these prior experimental studies, we have expanded the work to directly compare the findings to computational predictions for specific types of grain boundaries and their segregation. As these types of cross correlation microscopy experiments are difficult, the development of simulations that predict solute segregation to specific grain boundary types are needed.

A simulated grain boundary will require the full character of the boundary to be known, which can pose a challenge to several of the current experimental diffraction techniques. For instance, two- dimensional EBSD images provide three of the five parameters needed to fully define a grain boundary^23^. To achieve the missing parameters, the material must be serial sectioned to reveal the sub-surface grain boundary inclination angle^24^. Though similar restrictions can also exist in PED scans, recent work by Kiss *et al*.^25^,^26^,^27^ has shown how the inclination angle can be estimated by a weight average from the overlapping transmission diffraction patterns across the boundary. Moreover, one could also conceive using the APT data set volume itself to reveal the grain boundary topology^13^. This information could then be coupled to the prior diffraction of the boundary. However, the identification of the APT boundary surface necessitates particular reconstruction artifacts, such as a density variations, to be used to identify the boundary. Often these variations will not be uniform over the surface creating inconsistencies in the topology. If solutes are used is to identify the boundary surface, then those solutes also need to form a uniform coverage which may not be the case. These examples provide some of the experimental challenges one can encounter in providing the full character identification of an experimental boundary.

In this work, we detail our use of PED with the intrinsic morphology of a thin film columnar grain boundary to overcome those challenges such that the full character of the boundary could be revealed and linked to the simulations. Revealing that full character of the boundary is critical as recent simulation work has shown the importance of the inclination angle on grain boundary energies and motilities. For example, in body centered cubic metals, the ∑3 boundary energy was highly sensitive to the inclination angle whereas ∑5 were found to be more invariant^28^,^29^,^30^,^31^. Homer *et al*.^32^ have reported how the grain boundary inclination angle is sensitive to the mobility of equivalent ∑ boundaries even when those boundary energies were similar.

In this paper, using computational methods, we examine specific boundaries and how their grain boundary energies change with Cr solute segregation. Using that information, we compare the interfacial excess of those simulated ∑ boundaries to experimentally measured ∑ boundaries. By linking the experiment with these models, we are able to better understand the solute-boundary segregation behavior. These models then provide information on segregation over a larger compositional range without the need for the laborious experimental effort.

Our test material is a nanocrystalline Fe-8 at.% Cr thin film prepared by sputter deposition. We have recently reported how minor Cr additions to Fe can be used to change the as-deposited thin film stress state^33^. In that report, we described how the tensile stress varied with grain size with the grain size controlled by Cr segregation. Atom probe results in that work revealed a range of Gibbsian interfacial excess values for the boundaries studied which suggested boundary specific segregation behavior. However this was never confirmed and provided motivation for this new work. Besides thin films, larger scaled Fe-Cr systems are of strong technical interest because of their resistance to radiation effects, *e.g.* damage accumulation and swelling in future nuclear reactors^34^,^35^. Atom probe studies in these alloys has been done to address its spinodal decomposition^36^,^37^,^38^,^39^,^40^,^41^. Marquis’s group^42^,^43^,^44^,^45^,^46^ has shown that Cr segregation to specific grain boundaries does occur with respect to phase instability under irradiation conditions. Using EBSD, a specific grain boundary from a coarse grain material was extracted, FIB milled into the APT needle shape, and field evaporated in the atom probe. The results revealed irradiation enhanced Cr segregation to the ∑3 boundary with very little effect on low angle grain boundary (LAGBs) which were denoted as ∑1 boundaries^42^. Others have reported how Cr can desegregate from the boundaries after irradiation^47^,^48^.

## Experimental details

The case study material was deposited as a 300 nm thick Fe-8 at.% Cr film by co-magnetron sputtering from 99.95 at.% pure elemental Fe and Cr targets in an AJA ATC-1500 stainless steel chamber. Prior to deposition, the base pressure was <5 × 10^−8^ Torr where upon ultrahigh purity Ar was flowed into the chamber to 2 mTorr and served as the working gas. The film was grown on a Si [001] wafer which had a native surface oxide. After deposition, the film phase was identified to be a solid solution body centered cubic structure by X-ray Diffraction via a Philips diffractometer with Cu *K*_*α*_ radiation as the source operated at 45 keV and 40 mA.

**Figure 1:**
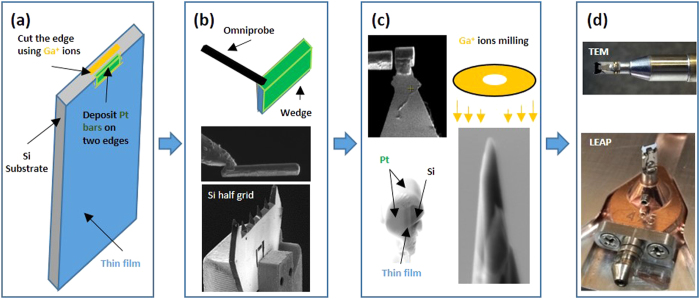
The procedure to prepare sample for correlative TEM and APT: **(a)** The bar with Pt protection on the top and left was cut by Ga^+^ ions; **(b)** The bar was lift out by Omniprobe. Si half grid into a hummingbird hold was also shown; **(c)** pieces of the bar were mounted to the top of Si half grid and milled and cleaned up by Ga^+^ ions using 30 kV and 5 kV, respectively; **(d)** The grid holder fitted directly into a hummingbird Tecnai F20 TEM single-tilt holder. Modification of a Cameca puck specimen assembly that allows the insertion of the hummingbird holder into the local electrode atom probe (LEAP) instrument.

The film was prepared into an atom probe tip by extracting the sample in cross section (parallel to the film-substrate interface) using FEI Quanta dual electron beam-FIB microscope. *In situ* Pt, using a Gas Injection System, was deposited over the film’s region of interest to serve as a protective coating during the FIB milling, as shown in Fig. 1(a). The extracted film wedge was then attached to a silicon half grid, Fig. 1(b)^49^, whereupon it was annular ion milled at 30 keV using a range of step down ion currents from 137 to 32 pA in a Tescan Lyra dual beam FIB, Fig. 1(c). When the final tip shape was approached (radius of curvature ~100 nm), the milling parameters were reduced to 5 keV 25 pA to reduce Ga^+^ implantation and surface damage. Further details of the sample preparation method can be found in refs 50 and 51.

With the APT tip prepared, the specimen was loaded into a Hummingbird TEM holder, Fig. 1(d), and placed into a FEI Tecnai F20 S(TEM) operated at 200 keV. The grain-to-grain mapping of the film was conducted using the precession electron diffraction (PED) technique from the Nanomegas ASTAR^TM^ platform^20^,^21^,^22^. By focusing the electron beam, a high spatial resolution for point-to-point diffraction imaging is achieved; coupled with precessing the beam, the dynamical diffraction effects are reduced allowing the diffracted intensities to be more uniform and kinematical in nature^20^. In addition, the precessed beam intersects more of reciprocal space increasing the number of diffracted reflections which increases the confidence for identifying the diffraction pattern. The collected patterns are indexed and the rotation between diffracted patterns used to quantify the grain boundary character using an off-line TSL OIM Analysis 7 software package. The estimated inclination angle of the grain boundary using the PED was done following the procedure by Kiss *et al*.^25^ with details specific to this work given in the supplementary material section. For these experiments, the beam was precessed at 0.1° at a scanning step size of 2 nm. After completing the PED scan, the tip was moved to a Cameca puck specimen assembly, Fig. 1(d), and field evaporated in a Cameca Instruments Local Electrode Atom Probe 3000 XSi operated with a specimen set point of 37 K, laser pulse energy of 0.3 nJ at a pulse repetition rate of 200 kHz for a 0.5% atoms per pulse detection rate. The atom probe data sets were analyzed using the IVAS 3.6 platform with the interfacial excess measurements done in a manner previously reported by the authors in ref. 33. Note, between the PED scan and the LEAP analysis, the APT tip was subjected to a brief, 5 keV Ga^+^ ion milling step to remove any surface and hydrocarbon damage created during the PED scan.

A TEM plan-view foil was also prepared by cutting, dimpling, and ion-milling a 3 mm disc. This foil provided a larger field of view of the nanocrystalline film to confirm phase, grain sizes, and grain boundary misorientations using the aforementioned NanoMEGAS PED platform. The TEM foil analysis provided a benchmark comparison to determine the representative nature of the boundaries quantified in the much smaller field of view provided by the APT tip. A TEM cross-sectional foil was also prepared by a FIB lift out technique with its grain boundaries quantified using PED. These results verified the columnar (linear) nature of the boundaries through the film thickness.

## Computational details

The computational simulations for twenty-seven types of ∑ boundaries were done to provide insights into solute segregation specificity using the methods in ref. 52. Of these twenty-seven boundaries, six specific type of boundaries were found in the experimental data set and provide the major discussion of the paper. The relevant information concerning the balance of these other boundaries are provided in the supplementary section. Of these six boundaries, several different orientation angles were done for each boundary type to determine the influence of inclination angle on the asymmetric boundary energy. The tilt grain boundary was created by rotating two BCC grains pertaining to a tilt axis, <uvw>, and joining the two grains by a grain boundary with the Miller index (hkl)^52^,^53^ creating a single boundary between the two lattices. The following relationship is required since the tilt axis and grain boundary plane must be parallel (1):


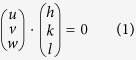


The (hkl) of the grain boundary plane is associated with the tilt angle, θ, with respect to the <uvw> tilt axis:


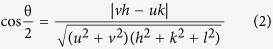


The minimum grain boundary structure was defined as rectangular cells that satisfied this periodic condition. The created CSL grain boundaries are given in Table 1, which have been relaxed using the Molecular Dynamics (MD) conjugate gradient minimization energy procedure. The work only minimizes a single structure. Though the selected grain boundary does not in of itself indicate the minimum energy structure, the simple structure of the boundary likely is the minimum or is a structure whose energy is closely related to the minimum energy structure as suggested by the work of Olmsted *et al*.^54^.

**Figure 2:**
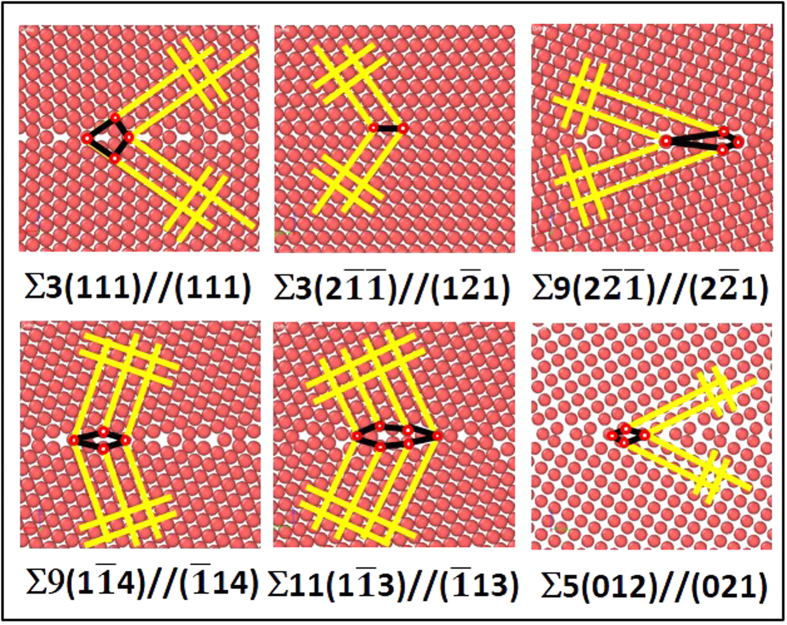
Structural views of CSL grain boundaries. The yellow lines guide the eye for the lattice structure whereas the black lines reveal the repeat structure of the boundary.

Figure 2 is a representation of part of these boundaries where the yellow lines serve as guides to identity the lattice and tilt angle; the black lines on either side of the boundary reveal the repeated atomic structure for that particular special character arrangement. The LAGB was constructed by tilting two grains with a small angle at either 4° or 8° which would technically classify them as a symmetric ∑1 boundary. This created a periodic edge dislocation spaced along the boundary with most of the lattice structure on either side of the boundary overlapping. This approximation is reasonable because of the low degree of coincidence associated with those CSL values. Since LAGBs have a range of misorientations, it is not reasonable to simulate every possible angle; hence we have averaged the findings between the values found in these specific LAGB simulations and reported the maximum and minimum values on the forthcoming simulated plots. The random high-angle grain boundary (HAGB) network was simulated using a polycrystalline structure based on ref. 55. Table 1 contains the tabulation of these and all ∑- constructed grain boundaries, with the ones bolded in the table highlighting the relevant boundaries that will be discussed in the paper (with the non-bolded boundaries found in the supplementary section).

Using these boundaries, we have implemented a progressive computation method that is a hybrid algorithm combining MD and Monte Carlo (MC) to determine the preferential segregation of Cr to these Fe grain boundaries^56^. The code was run using LAMMPS^57^ with the Fe-Cr binary concentration dependent embedded atom model potential found in ref. 58. To change the Cr content, we adjusted the system’s chemical potential until the Cr content matched the targeted composition. We performed both a variance-constrained semi-grand-canonical ensemble and a semi-grand-canonical ensemble and noted that the results were similar for both methods over the compositions range from 0–14 at.% Cr. One MC cycle was carried out per 10^2^ MD steps. The equations of motion were integrated for 10^5^ MD steps, which included the 10^2^ MC cycles, using a time step of 2 fs. Temperature and pressure were maintained using the Nose-Hoover thermostat and barostat, respectively.

## Results and Discussion

**Figure 3:**
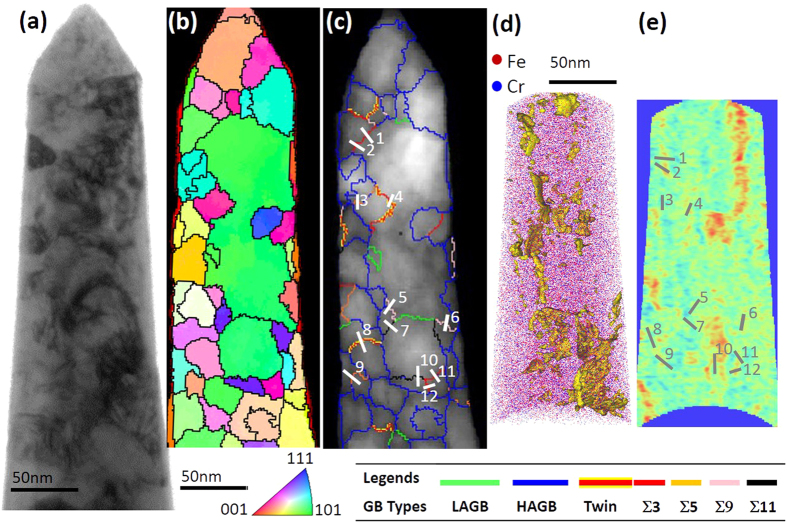
(**a**) TEM image of the Fe 8 at.% Cr atom probe tip **(b)** PED orientation map of the same atom probe tip **(c)** CSL boundary line map laid over the image quality map of the tip; **(d)** Atom map image with grain boundaries delineated by an isodensity surface **(e)** 2D composition profile map with grain boundary sequences overlaid.

Figure 3 is the correlative bright field TEM image [Fig. 3(a)] with the PED grain orientation map [Fig. 3(b,c)] as well as various atom probe map reconstructions [Fig. 3(d,e)] from the APT specimen. Electron diffraction confirmed that the alloy was a solid solution, body centered cubic, A2, phase. The reconstructed atom map contained over 47 million ions collected from a 220 nm vertical length. By comparing the isoconcentration reconstruction, Fig. 3(e), to the PED quality index boundary map, Fig. 3(c), we are able to directly correlate the composition to the specific grain boundary character. From the boundary map [Fig. 3(c)], we identified twenty-three grain boundaries with either LAGBs (2 GBs), HAGBs (9 GBs), or one of the five special character boundary types – ∑3(twin) (2 GBs), ∑3 (4 GBs), ∑5 (2 GBs), ∑9 (2 GBs), and ∑11 (2 GBs). The positions of special character boundaries were marked in Fig. 3(c,e).

To determine if the ∑3 was or was not a twin involved two criteria. First, the misorientation across a grain boundary must be very near the twin misorientation, which was readily identified by the OIM reconstruction software from the PED patterns. The second criteria involved the boundary plane coinciding with the twin plane. Though this is more difficult (if not impossible) to identify in two-dimensional projections, one can assess whether the trace of the boundary plane is aligned with the trace of the twinning plane as a partial check. When that occurs, the boundary, approximately 90% of the time, is a twin^59^. This provided a fairly high confidence means in twin identification. Subsequently, the twins were also confirmed by a comparison of their deviation from the symmetric plane position taken from the PED scan itself in our inclination angle determination. This, and all other boundaries, inclination angles are tabulated in Table 2.

**Figure 4:**
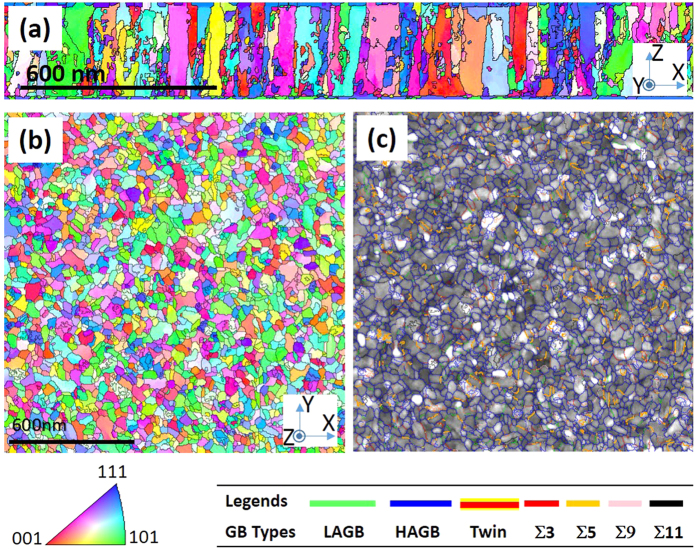
(**a**) Cross-sectional and **(b)** Plan-view PED orientation map with **(c)** Different grain boundary lines laid over the image quality map for the Fe_0.92_Cr_0.08_ film.

The inclination angle was determined by either measuring the PED projected boundary by the method in ref. 25 and/or assuming that the boundary itself was perpendicular to the plane normal when the PED scan was inconclusive. This latter assumption was confirmed by the TEM cross sectional view, Fig. 4(a), which showed the columnar morphology of the boundary. To validate that the APT tip is representative of the specimen as a whole, a PED scan over a planar TEM foil capturing over 800 grains, Fig. 4(b,c), was performed. Though the APT analysis region is smaller, we noted the same grain boundary types with good agreement with respect to their fractional lengths in the material between the two PED scans, Table 3.

With the grain boundaries now identified in the atom probe data set, the experimental Gibbsian interfacial excess, Γ_Cr_, of Cr solute at those boundaries was calculated^60^ and tabulated in Table 2. The HAGBs, followed by ∑3, revealed the highest concentration of solute species than any other boundary. Our findings are consistent with prior reports of other solute segregates in Fe-based alloys by Herbig *et al*.^61^ and Hu *et al*.^42^, who also noted a solute segregation preference to ∑3 boundaries.

From the correlated PED-APT data set, it is clear that preferential partitioning of Cr to specific grain boundaries has occurred. To understand this boundary specific segregation behavior, as well as survey the effect of Cr segregation over a larger compositional range and boundary types, the hybrid MD/MC simulation was performed.

**Figure 5:**
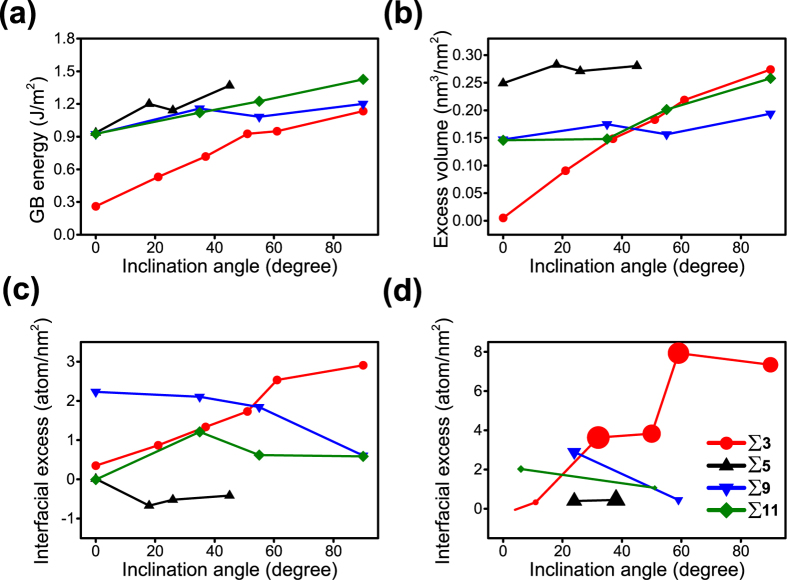
(**a**) Calculated grain boundary energy **(b)** Calculated excess volume **(c)** Calculated and **(d)** Experimentally measured interfacial excess for ∑3, ∑5, ∑9 and ∑11 asymmetric GBs as a function of inclination angle. The symbol size in (**d**) was in direct proportion to the deviation of the values measured for those particular misorientation angle for those GBs.

Figure 5(a) is a series of plots of simulated Cr segregation behavior for the Fe-8% Cr alloy at various inclination angles. The ∑3 grain boundary energy appears to be the most sensitive to inclination angle. As the inclination angle increased, so did the grain boundary energy, Fig. 5(a), which is consistent with prior reports^32^. The other special character boundaries – ∑5 and ∑11 – also showed some modest increase in energy with inclination angle in contrast to ∑9, which was relatively invariant over the inclination angle range.

Since segregation could be influenced by the reduction of excess volume in the boundary by Cr, we have also explored the change in grain boundary excess volume and the solute energetic distribution in those special character boundaries^53^,^62^. In the case of the volume excess change, the Cr atom is a slightly larger atom than Fe^63^ where one can expect that Cr at the grain boundary would reduce the excess grain boundary volume. Hence, the volume excess, *V*_*excess*_, was computed as


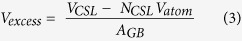


where *V*_*CSL*_ is the simulated volume that contains the CSL boundary, *N*_*CSL*_ is the number of atoms in total volume, *V*_*atom*_ is the average atomic volume for a specified composition but with no CSL boundary, and *A*_*GB*_ is the surface area of the CSL grain boundary in the simulated volume. Figure 5(b) is a plot of how that volume would change with inclination angle for each of the identified boundaries in this study.

Comparing Fig. 5(a,b), similar trends can be noted, with the excess volume increasing the most for the ∑3 boundary for the Fe-8% Cr alloy. However, of all the boundaries, only the ∑3 shows a clear increase in the interfacial excess of Cr with increasing inclination angle, Fig. 5(c). ∑11 predicts a modest increase and then decrease in interfacial excess initiating near 30° inclination; ∑5 revealed a strong preference for rejecting Cr from its boundary regardless of inclination angle; and ∑9 indicated a reduction of Cr concentration in its boundary with increasing inclination angle. Of all ∑ boundaries, ∑3 provides a consistent trend between boundary energy, increase in excess volume, and increase in solute concentration with increases in the inclination angle. This suggests that as this boundary becomes more asymmetric, changes in Cr segregation will be the most sensitive (noticeable).

Figure 5(d) is a plot of the experimentally determined excess for each of the boundaries quantified by the cross correlation microscopy. Comparing these findings with the predicted interfacial excess in Fig. 5(c), the trends as a function of inclination angle are in good agreement, with ∑3 showing an increase in interfacial excess with inclination angle; ∑5 being invariant and very low (near zero); and ∑9 and ∑11 revealing a decrease in solute concentration with increasing angle. Considering the limited number of boundaries that could be captured within the finite field of view of the APT reconstruction, the good agreement with the predicted trends adds confidence in the more limited experimental findings.

**Figure 6:**
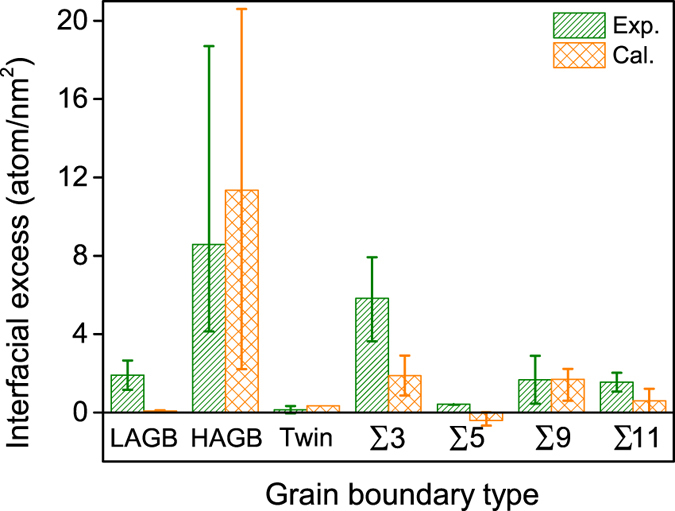
Experimental and simulated interfacial excesses varying with grain boundary types. Error bars represent upper and lower limits. Cal. (calculated) refers to the simulated boundaries.

Though the trends were in good agreement, in general, all of the boundaries did reveal slightly higher absolute values in the experimental interfacial excess than predicted values; the exceptions being the ∑9 and ∑11 at the higher inclination angles where close agreement with the predicted values was seen. The most dramatic differences in absolute values was noted for the ∑3, being nearly 2.5 times larger. Though the exact reasoning for this difference is not fully understood, particularly considering consistent trends between the two studies as well as some limited boundaries having a much closer agreement with each other, one potential source could be the thin film process itself. During deposition, the segregation to those boundaries had not achieved the lowest energy state since they were not annealed. During the dynamic growth of the Fe(Cr) film, as the grain boundaries developed during film coalescence, it is possible that excess Cr was likely incorporated within some of these special character boundaries. Computational modeling the effects of solute segregation during film growth is the subject of ongoing work by the authors and is beyond the scope of the current work. Regardless of the absolute value difference, the experimental values are realistic and within the correct order of magnitude of the predictions and mimic the predicted trends added confidence in linking the two studies. For easier comparison, a histogram of the experimentally measured and computationally predicted Γ is plotted in Fig. 6, with the addition of both the low and high angle grain boundaries, which will be discussed in further detail below.

**Figure 7:**
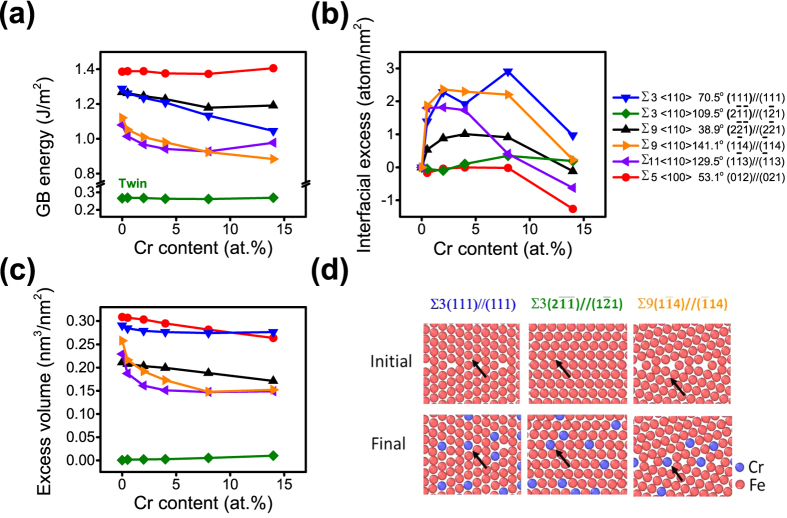
(**a**) Calculated grain boundary energies **(b)** Interfacial excess and **(c)** Excess volume for various Cr contents for specific ∑-GBs. **(d)** Simulated atomic configuration for ∑-GBs with and without Cr segregation. The arrows direct to local lattice locations where relaxation from the initial to final states can be tracked.

To expand our computational study, we have also looked at the segregation as a function of Cr content for specific boundary constructions, ∑3(111)//(111), ∑3(2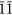)//(1
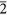
1) (twin boundary), ∑9(2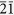)//(2
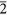
1), ∑9(1
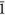
4)//(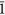
14), ∑11(1
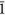
3)//(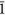
13) and ∑5(012)//(021). Figure 7(a) is a plot of grain boundary energy with increasing Cr content, where the energy values for the majority of these boundaries were reduced^64^; the exceptions being the ∑3(2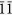)//(1
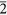
1) (twin boundary) and ∑5 boundaries, Fig. 7(a). The Cr is predicted to have discernable segregation to all of those represented boundaries expect, again, the twin and the ∑5, which were nearly invariant, Fig. 7(b). Recall that ∑5 was also relatively invariant in response with inclination angle for Fe-8% Cr composition, Fig. 5. For the boundaries where segregation was predicted, the interfacial excess was the largest at the lower Cr contents. In all of these cases, the amount of grain boundary energy reduction (and segregation tendency) was not found to be equivalent for each boundary type. This was similar to the inclination study previously discussed; where the ∑5 and ∑9 grain boundary energy increased or was relatively consistent, respectively, with inclination angle but the interfacial excess was invariant or decreased respectively for each of these boundaries. In these new studied boundaries, the ∑3(111)//(111) appeared to have a monotonic reduction in energy with increasing Cr content whereas ∑9(2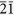)//(2
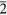
1) revealed an initial decrease with a ‘leveling-out’ of the energy with Cr content, Fig. 7(a).

As before, we have applied equation (3) to compare how these boundary energies and segregation scale with the change in excess volume created by the segregation of Cr. The excess volume change with Cr content is plotted in Fig. 7(c). The ∑3(2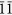)//(1
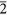
1) twin did not show any significant change in excess volume. A very limited free volume change would be expected since this special boundary has a high frequency of coincident lattice sites overlapping each other at the boundary. The near invariant change in excess volume is similar to the equivalent invariant change in grain boundary energy, Fig. 7(a). Hence, no substantial energy gain would occur by the placement of Cr into this boundary. This is schematically shown in Fig. 7(d), where the boundary structure does not change and is in agreement with the predicted and experimentally measured interfacial excess found for the twin boundary in Fig. 5(c,d), with Γ ~ 0 atoms/nm^2^. In contrast, the excess volume reduction trends in Fig. 7(c) for ∑11(1
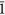
3)//(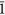
13), ∑9(1
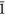
4)//(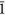
14), and ∑9(2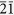)//(2
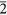
1) did show relative agreement with how the grain boundary energy changed with Cr content in Fig. 7(a). This could suggest that a driving force for Cr segregation to these boundaries is linked to the reduction in excess volume. In Fig. 7(d), the Cr segregation to the ∑9(1
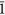
4)//(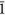
14) is shown with the arrow to lead the eye to view where the boundary relaxation occurred.

Interestingly, the ∑3(111)//(111) and ∑5(012)//(021) do not show similar trends between grain boundary energy and excess volume behavior with Cr content. This discrepancy between trends gives insights into the subtle and complex differences that regulate grain boundary segregation as a function of boundary type. In the case of the ∑3(111)//(111), similar to its twin counterpart (∑3(2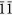)//(1
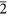
1)), the ∑3 CSL boundary has a high frequency of overlapping lattice sites at the boundary, Fig. 7(d). As before, one could infer that a reduction of excess volume would be minimal for this particular type of special boundary. However, the ∑3(111)//(111) has a higher interfacial excess than its ∑3(2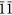)//(1
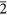
1) twin. This difference in segregation between the ∑3 boundaries likely resides in one being coherent and the other being incoherent.

**Figure 8:**
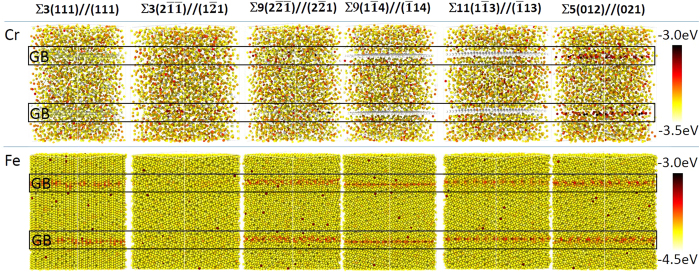
Cr or Fe atomic energetic distribution for different types of ∑-GBs. The GB is highlighted by the rectangular box.

To explore this concept further, the energetic distribution for the atoms in and away from these specified boundaries are shown in Fig. 8 for the Fe-8at.%Cr alloy. In this figure, the top and bottom row of images are the Cr and Fe atoms respectively. The color variation within each row corresponds to potential energy of that atom (Cr or Fe) in the matrix or at the boundary. For the ∑3(111)//(111), the Fe atoms in the boundary has a higher energy as compared to those in the matrix, evident by the linear cluster of red spheres in the boundary. In contrast, the Cr atoms in the same boundary appeared to have a lower energy. Comparing this to the twin ∑3(2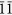)//(1
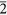
1), which revealed no discernable energy differences in or away from the boundary for either Fe or Cr, suggests that the chemical nature of the incoherent boundary appears to influence the Cr segregation.

In the case of the ∑5(012)//(021), the grain boundary energy is relatively invariant with Cr content, Fig. 7(a), but a modest reduction in excess volume with Cr segregation is predicted, Fig. 7(c). One could speculate that a reduction in excess volume at this boundary would promote segregation and manifest itself with a reduction in grain boundary energy. However the Gibbsian interfacial excess is at or near zero up to ~8 at.% Cr, Fig. 7(b), demonstrating no clear thermodynamic preference for Cr. The energetic distribution, Fig. 8, reveals a higher energy for these Cr atoms to be at this ∑5(012)//(021) boundary evident by the linear collection of red spheres at the boundary. It is interesting to note that this is the only boundary studied that showed such a distinct (red) energy value for Cr at a boundary. Upon increasing the Cr content above this value, the interfacial excess, Fig. 7(b), becomes negative indicating a strong tendency for this boundary to reject Cr. Collectively, these results reveal the Cr is not preferred at ∑5(012)//(021), which was also seen experimentally albeit at a ∑5 with a different inclination angle.

The most favorable energy state for Cr to be located in the boundary is seen for the ∑9(1
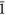
4)//(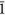
14) and ∑11(1
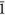
3)//(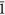
13), with a linear cluster of white spheres at these boundaries. The corresponding Fe were noted to be red (having a higher energy) in these boundaries. This is in agreement with the interfacial excess behavior, Fig. 7(b), which showed favorable segregation. The rapid increase of these excess values at lower Cr concentrations is likely associated with highly favorable energetics for Cr segregation to these particular boundaries with their excess volume reduction at those particular concentrations.

**Figure 9:**
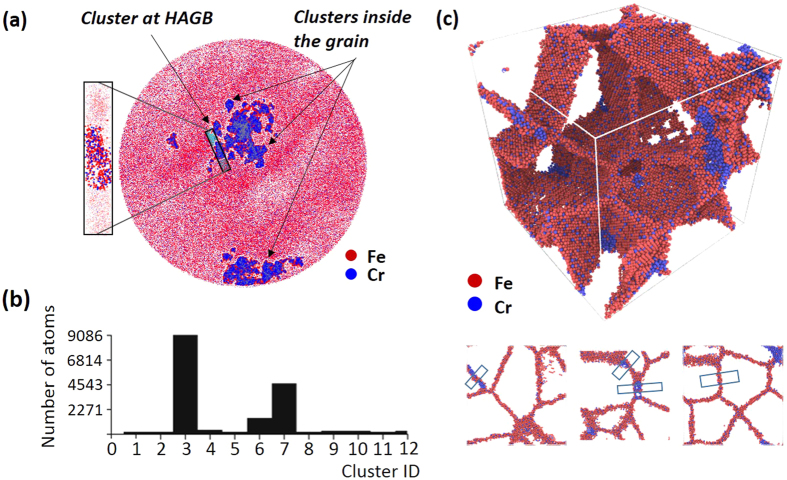
(**a**) Experimental result of cluster analysis laid over a 10 nm thick atom map (top view) extracted from the original APT data set **(b)** The histogram of cluster size from (**a**). **(c)** Simulation result of clusters forming at polycrystalline GBs with selected images of the clustering at the boundaries shown below the volumetric rendering of all the boundaries.

Returning to Fig. 6, we now address the low and high angle grain boundaries. For the LAGBs, experimentally, the interfacial excess is higher than what was predicted. A low interfacial excess would likely be expected since these boundaries have very good lattice alignment between the grains^65^. And in particular, the simulation was based on a symmetric tilt GB was used which may not properly capture the true experimental LAGB structure. In contrast, the high interfacial excess for both the experimental and simulated Cr segregations in the HAGBs is suspected to be associated with Cr’s clustering. As shown in Fig. 9(a,b), experimentally identified clusters were observed in these boundaries. These clusters were defined in the reconstruction to have a threshold value of 20 at.% Cr or higher within a predefined spherical sampling volume of 1 nm^3^. The vast majority of these clusters were noted in the HAGBs (whose boundaries were verified by the prior cross-correlated PED scan of the APT tip). The balance of the other clusters (which were few) were observed to be within the grains themselves and no clusters were noted in the other types of boundaries. This clustering behavior is believed to be the early onset of phase separation, which has been similarly reported in prior APT studies for various aged Fe(Cr) specimens^36^,^66^. The onset of clustering, even in the sputter deposited film, could be expected since this alloy’s composition (~8 at.% Cr) exceeded the solubility limit of Cr in Fe at room temperature (~5 at.% Cr)^34^. Since HAGBs provide pathways for rapid diffusion^67^ coupled with larger excess volumes and higher grain boundary energies as compared to special character boundaries^68^, they provide a favorable kinetic and thermodynamic site to initiate the precipitation of the Cr phase. To further corroborate this behavior, a polycrystalline simulation of a HAGB network was performed.

We initiated this polycrystalline simulation by using the experimental composition of Fe-8 at.% Cr. Unfortunately, we were unable to identify similar clusters at this overall composition. This was contributed to the limitations of the simulation time to initiate clustering for this particular composition. However, by increasing the Cr content to 14 at.% within our available simulation time, clustering was detected. As seen in Fig. 9(c), the simulated clusters were observed and compared to the experimental APT boundaries in Fig. 9(a). The interfacial excess values for these simulated boundaries are plotted in Fig. 6. Similar to the experimental findings for the HAGBs, the Gibbsian excess value’s standard deviation was large. This larger excess value spread is contributed to the dilute and highly enriched Cr within the boundary and between the different clusters. Hence, depending on where the interfacial excess is measured, the values would vary considerably and is manifested in the larger error around the average value. Hence, in both the simulation and experimental APT data, the relative amount of interfacial excess was then dependent on the size and location of the cluster with values ranging from a few to tens of atoms/nm^2^. This simulation confirmed that HAGBs were a preferred segregation boundary in the alloy where clustering occurred as compared to the other identified special character boundaries.

## Conclusions

The comparative simulations with correlated TEM and APT has provided experimental verification of grain boundary type dependencies for segregation. The experimental interfacial excess values are similar to the predicted values and exhibit the equivalent trends in segregation to certain types of ∑-boundaries. This provided confidence in simulated understanding and predictions of segregation in Fe(Cr) for other types of specific boundaries.

Our findings confirm that ∑3 boundaries are most sensitive to inclination angles changes. This resulted in a larger spread in Cr interfacial excess values, both experimentally and predicted by the simulation. Both the experiments and simulations revealed similar trends in interfacial excess values for the other special character boundaries, though, in general, the experimental absolute values were slightly larger. Of all the types of grain boundaries studied, the HAGBs had a higher Cr solute concentration. This was contributed to these features being preferred sites for clustering and the eventual initiation sites for Cr precipitation, which was confirmed by simulations. The direct and consistent linkage of experimental and simulation findings for the Fe-8% Cr alloy gave confidence in the simulation predictions.

Consequently, the segregation behavior of Cr from 0 to 14 at% was undertaken for other types of specific grain boundaries. The solute-boundary segregation behavior was then discussed in terms of both volume excess reduction and the energetic distribution of Fe and Cr in and away from the boundaries. For the special boundaries, the twin ∑3(2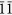)//(1
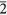
1) and ∑5(012)//(021) grain boundary energies were relatively invariant to the Cr content with little to no segregation of Cr to their boundaries. This was in agreement with the prior experiments. In contrast the ∑3(111)//(111) had the highest interfacial excess, for all special character boundaries, with a linear decrease in grain boundary energy with increasing Cr content. The ∑9(1
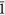
4)//(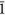
14), ∑9(2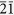)//(2
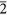
1), and ∑11(1
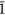
3)//(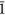
13) revealed a positive interfacial excess, though at a lower positive value, and a reduction of grain boundary energy whose change was more pronounced at lower Cr contents.

The evidence of specific grain boundary segregation, even in an as-deposited room temperature film, demonstrated the energy preference for grain boundary specific segregation. The added simulations have provided insights into why specific boundaries are more or less favorable for this segregation.

## Additional Information

**How to cite this article**: Zhou, X. *et al*. Grain Boundary Specific Segregation in Nanocrystalline Fe(Cr). *Sci. Rep.* **6**, 34642; doi: 10.1038/srep34642 (2016).

## Supplementary Material

Supplementary Information

## Figures and Tables

**Table 1:** Structural information for the various CSL GBs studied.

Type	Tilt axis	GB plane	Tilt angle	Inclination Angle	No. of atoms
**∑3**	**<110>**	**(111)//(111)**	**70.5°**	**90°**	**51840**
**∑3 Twin**		**(2** 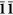 **)//(1** 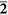 **1)**	**109.5°**	**0°**	**55296**
**∑9**		**(2** 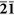 **)//(21)**	**38.9°**	**90°**	**54432**
		**(1** 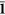 **4)//(14)**	**141.1°**	**0°**	**51840**
**∑11**		**(13)//(13)**	**129.5°**	**0°**	**57024**
**∑5**	**<100>**	**(012)//(021)**	**53.1°**	**45°**	**56160**
**∑801**	**<110>**	**{1 1 40}**	**4.0°**	**0°**	**57600**
**∑201**		**{1 1 20}**	**8.1°**	**0°**	**57600**
**∑3**	**<110>**	**(1** 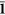 **1)//(** 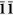 **5)**	**109.5°**	**21°**	**62150**
		**(100)//(22)**		**37°**	**54416**
		**(55)//(711)**		**51°**	**66066**
		**(110)//(411)**		**61°**	**51814**
**∑9**		**(11)//(15)**	**141.1°**	**35°**	**62208**
		**(1** 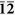 **)//(5** 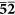 )		**55°**	**58320**
**∑11**		**(5** 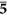 **4)//(18)**	**129.5°**	**35°**	**71280**
		**(4** 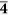 **1)//(2** 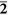 **5)**		**55°**	**76032**
		**(3** 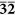 **)//(3** 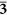 **2)**		**90°**	**63360**
**∑5**	**<100>**	**(031)//(03** 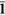 )	**36.9°**	**0°**	**58240**
		**(010)//(04** 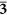 )		**18°**	**52000**
		**(110)//(071)**		**26°**	**62400**
∑7	<111>	(3 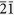 )//(2 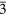 1)	38.2°	—	50400
∑13a	<100>	(023)//(032)	22.6°	—	56784
∑15	<210>	(1 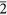 5)//( 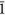 25)	48.2°	—	57600
∑17a	<100>	(041)//(04 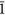 )	28.1°	—	53040
∑17b	<221>	(3 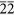 )//(2 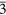 2)	61.9°°	—	48960
∑19a	<110>	(3 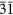 )//(3 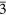 1)	26.5°	—	54720
∑19b	<111>	(5 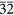 )//(3 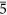 2)	46.8°	—	58368

The first group are the symmetric GBs, the second is the LAGBs, the third is the asymmetric tilt ∑GBs, and the last un-bolded designation are the GBs discussed in the supplemental material, as those misorientation were not observed experimentally.

**Table 2:** List of GB characters and the corresponding interfacial excess data for the experimental CSL GBs investigated.

Sigma	No.	Mis-orientation	Deviation	Rotation axis	Orientation (1)	Orientation (2)	GB planes	Inclination angle (REF)	Inclination angle (PED)	IE (atoms/nm^2^)
∑3(Twin)	08	59.3°	0.7°	[ 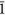 1 1]	( 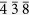 )[1 28 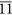 ]	(2 1 5)[9 7 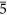 ]	[7 25 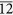 ]//[ 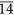 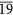 10]	4°	5°	−0.05
	04	59.6°	2.2°	[13 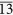 12]	(19 2 20)[8 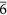 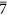 ]	(5 4 20)[ 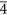 15 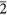 ]	[12 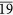 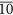 ]//[ 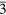 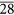 6]	11°	15°	0.33
∑3	02	59.5°	7.2°	[ 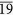 16 14]	( 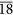 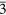 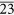 )[ 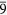 8 6]	(14 1 16)[6 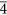 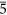 ]	[ 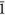 19 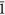 ]//[ 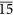 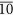 14]	32°	16°	3.63
	01	59.5°	7.2°	[ 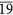 16 14]	( 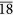 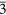 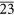 )[ 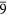 8 6]	(14 1 16)[6 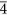 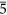 ]	[ 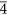 13 2]//[ 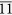 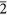 10]	50°	9°	3.83
	11	58.0°	8.4°	[18 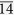 13]	( 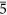 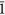 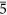 )[ 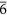 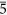 7]	(7 6 26)[10 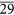 4]	[ 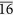 1 16]//[ 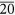 17 1]	59°	—	7.93
	12	59.6°	6.1°	[4 5 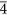 ]	(1 1 4)[2 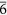 1]	(8 8 11)[ 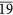 41 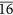 ]	[ 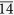 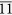 6]//[ 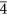 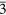 5]	90°	—	7.34
∑5	09	36.0°	5.4°	[ 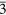 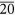 0]	(15 5 18)[ 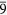 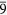 10]	( 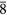 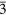 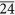 )[15 24 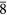 ]	[ 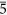 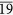 10]//[ 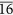 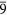 6]	24°	—	0.40
	03	34.4°	6.4°	[0 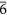 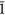 ]	(3 1 3)[ 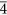 9 1]	(1 1 5)[ 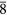 23 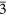 ]	[ 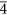 9 1]//[7 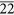 3]	38°	22°	0.45
∑9	06	41.3°	4.6°	[ 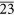 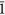 19]	( 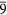 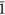 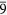 )[11 9 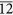 ]	(2 2 3)[ 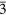 6 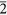 ]	[11 18 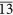 ]//[5 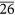 14]	24°	—	2.89
	05	38.7°	3.2°	[ 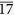 0 20]	(8 1 8)[11 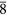 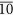 ]	(19 17 25)[ 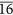 37 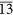 ]	[5 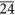 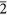 ]//[ 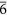 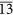 14]	59°	—	0.45
∑11	10	49.5°	2.4°	[10 0 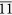 ]	( 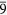 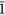 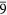 )[ 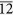 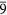 13]	(8 8 11)[ 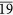 41 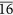 ]	[ 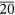 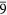 21]//[6 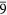 2]	6°	19°	2.03
	07	49.1°	1.7°	[ 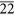 21 0]	(5 5 7)[ 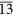 27 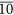 ]	( 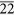 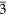 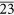 )[ 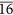 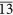 17]	[ 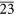 17 5]//[14 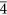 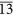 ]	51°	—	1.07

The No. value represents where that boundary is located in reference to Fig. 3. The misorientation, deviation, rotation axis, and the orientation (1) and (2) (for the grains), and the GB planes are extracted directly from the reconstructed PED data using the OIM software. The inclination angle “REF” refers to reference inclination angle as described in the supplementary section and the inclination angle “PED” refers to measured values from the method found in the paper of Kiss *et al*.^25^ also detailed in the supplementary section. Finally the interfacial excess, IE, for each to the measured special character boundaries is given.

**Table 3:** Fraction of grain boundaries in the APT tip and planar TEM foil.

Type		LAGB	HAGB	∑3(Twin)	∑5	∑9	∑11
Fraction	APT Tip	5.9%	75.3%	9.4% (4.6%)	3.1%	3.5%	2.8%
	Planar foil	5.2%	74.1%	11.0% (5.2%)	3.4%	2.9%	3.4%
