# Supplementary material for: Grain Boundary Specific Segregation in Nanocrystalline Fe(Cr)
Source: Sci Rep. 2016 Oct 6;6:34642. doi: 10.1038/srep34642 (PMC5052592; doi:10.1038/srep34642)
Supplement: Supplementary Information [file srep34642-s1.pdf]

**Supplementary Material for:**

# Grain Boundary Specific Segregation in Nanocrystalline Fe(Cr)

Xuyang Zhou[1], Xiao-xiang Yu[1], Tyler Kaub[1], Richard L. Martens[1], and Gregory B. Thompson[1*]

[1]The University of Alabama, Department of Metallurgical & Materials Engineering, Box 870202, Tuscaloosa, AL 35401-0202

*Correspondence to: gthompson@eng.ua.edu

1. Quantifying the experimental grain boundary normal and inclination angle:

To measure the inclination angle, we utilized the technique reported by Kiss *et al.*[1]. This method measures the inclination angle based on the projected width of the boundary as determined by the overlapping Precession Electron Diffraction (PED) patterns that would occur as the beam rasters over the boundary, i.e. whenever the grain boundary plane is oriented oblique to the electron beam, the overlapping area of neighboring grains can be discerned. The projected width of the GB is then measured using the following equation

$$\tan\omega = \frac{d}{t} \quad \text{(S1)}$$

where *d* is the projected width of grain boundary and *t* is the thickness of the sample, as shown in Fig. S1. For our work, the thickness refers to the diameter of the APT tip. The experimental GB inclination, Fig. S2(a)-(b), was determined by plotting the weight-factors of each diffracted intensity contribution from either grain on each side of the boundary, Fig. S2(c) and (d). As the beam rastered closer to the other grain, more of the diffracted pattern from that grain will be observed in the pattern. As that pattern becomes more pronounced, it is weighted more. IN this method, one can then quantify the diffraction pattern differences in overlapping patterns which

can then be used to estimate the GB inclination. The average distance between the two fitted lines in Fig. S2(c) and (d) defines the projection width of the grain boundary, albeit based on the resolution of the electron probe, which was ~ 2.5 nm. In the case shown, the width was approximately 16 nm with a scanning step size of 2 nm. Assuming that the diameter of the APT tip was approximately 100 nm (based on the TEM image, Fig. 3(a)), the experimental inclination angle is approximately 9$^{o}$. Due to the experimental limitations of either a very fine grain sizes and/or low image quality factors for the PED pattern captured, it was not possible to provide the inclination angle for each and every grain. In those cases, as mentioned in the paper, we have assumed that the grain boundary plane is parallel to the electron beam or, alternatively, we assumed no inclination to the boundary in the viewed projection. This was a reasonable assumption based on the cross-sectional TEM image, Fig. 4(a). The inclination angles that were measured are tabulated in Table 2, under the column 'Inclination angle (PED)'.

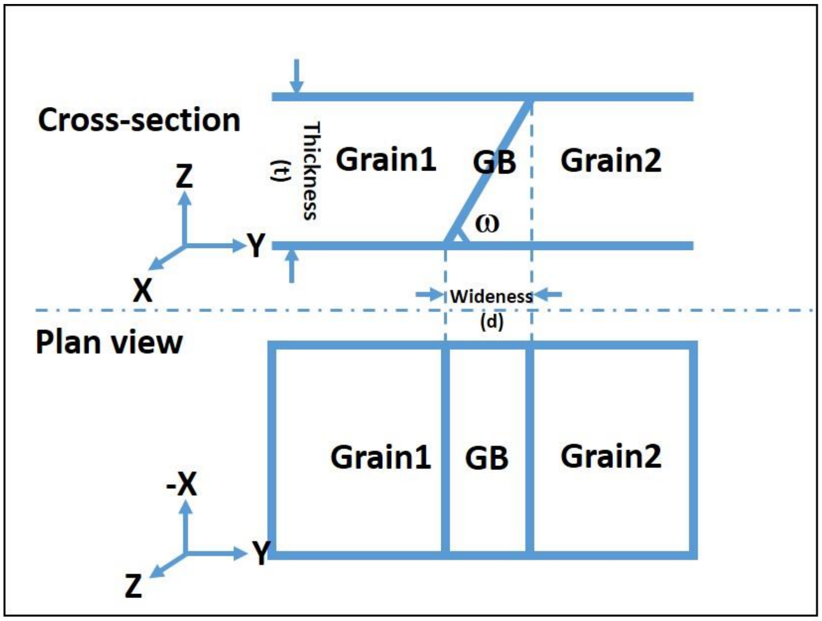


**Fig. S1:** Schematic of the cross section and projected boundary with designated variable to define the boundary width and inclination from a PED scan.

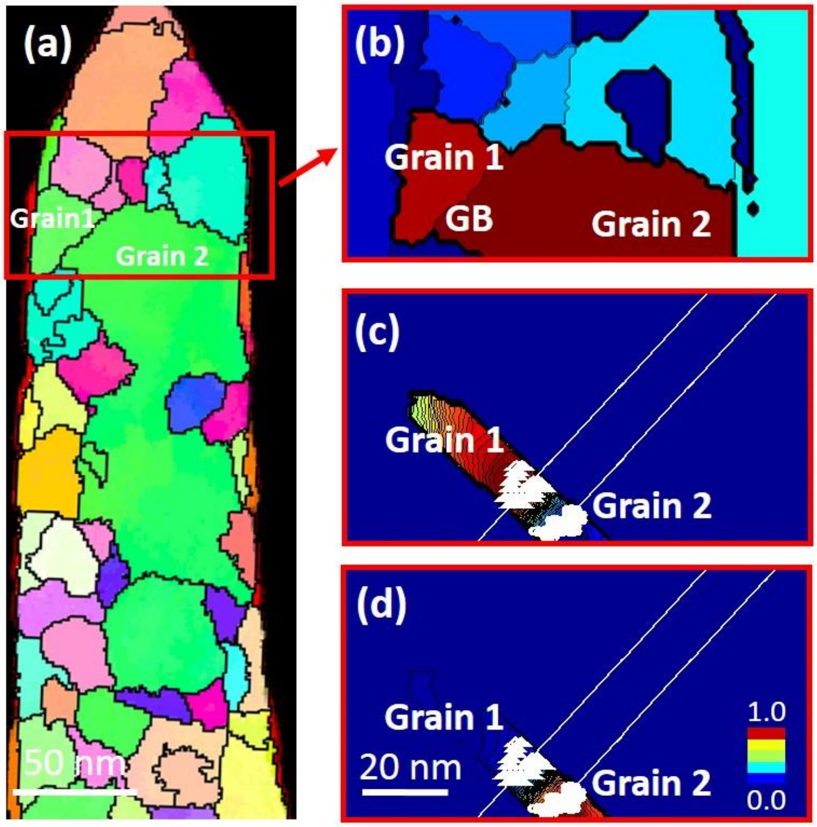


**Fig. S2: (a)** Orientation map of the APT tip (Fig. 3(b)) **(b)** Magnified image of a region of interest from (a) **(c)** the change in diffracted intensity (weight factor) of Grain 1 as it approaches the grain boundary projection, indicted by the white line **(d)** the change in diffracted intensity (weight factor) of Grain 2 as it approaches the grain boundary projection, indicted by the white line. The white marked features represent the weight factor values used to fit the projected boundary line.  The method for (c) and (d) were taken from Kiss *et al*.[1]

2. Determining a common reference system for inclination angles – computed and measured:

Though the experimental grain boundary inclination angle is important, it does not necessarily provide the common reference plane from which the inclination angle relative to the two fixed lattices can be compared.   In order for one to determine this inclination angle, a reference plane must be defined.

## 2.1 Reference inclination angle for simulated grain boundaries

For the simulated images, this was done by establishing the position within the lattice from which the angle of inclination, $\Theta$, to the GB plane's projection can be measured. To determine the grain boundary inclination angle an appropriate and common viewing direction based on the CSL type is required. For example, we used the [110] viewing direction for the $\Sigma3$, $\Sigma9$, and $\Sigma11$ and [100] viewing direction for $\Sigma5$. The choice of viewing direction was largely based on our ability to readily identify a series of parallel planes between each grain that would form the grain boundary between the two grains.

With the grain boundary now defined by the two sets of parallel planes in each grain, the reference plane was then required. This was done by defining a unit cell in each grain after which the symmetric plane between the two grains could be determined. This symmetric plane then served as the reference plane to the grain boundary plane created by the parallel planes from the viewing direction. Using the reference plane and grain boundary plane, the grain boundary inclination angle can then be measured.

These concepts are illustrated in Fig. S3 for an asymmetric $\Sigma3$ boundary. Here, the boundary is being viewed along the [110] direction for BCC Fe. The grain boundary, represented by the projected orange line, was formed by the two parallel planes listed in the figure, i.e. $(55\bar{1})$ and $(711)$. After which, the repeating unit cell in each grain, highlighted by the green boxes, was identified along with the symmetric plane between these two unit cells. This is graphically illustrated by the projected blue line in Fig. S3. The angle of inclination was then measured from the blue projected line to the projected orange line.

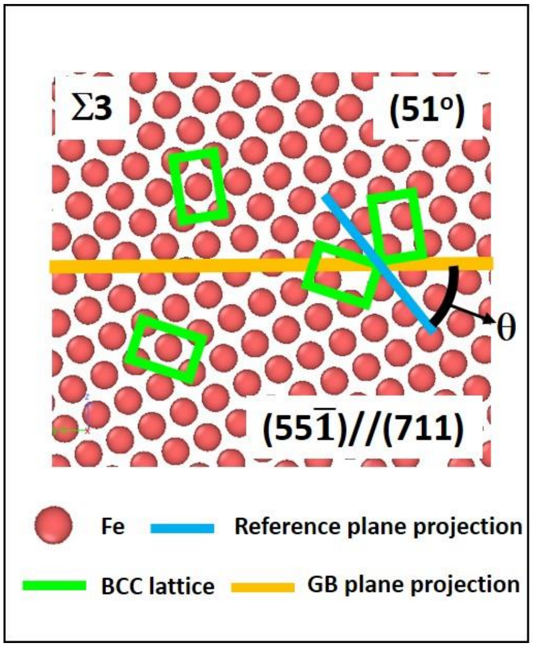


**Fig. S3**: A simulated inclination angle measurement from a Σ3 boundary created by the abutment of (55$\bar{1}$) parallel with (711). The green lines guide the eye for the unit lattice structure whereas the orange line is a projection of the GB plane and the blue lines being the projection of the symmetric plane between the two unit cells. The measurement of the blue projection line and orange projection line provides a common reference position of the inclination angle, Θ, determination.

In Fig. S4, four types of simulated Σ boundaries are shown, with the theoretical inclination angle changed from $0^o$ to $90^o$. For Σ5, we only titled to $45^o$ because above this angle, the conditions are symmetric to the values below this angle. Using specific tilt angles, we then were able to calculate the grain boundary energy, excess volume, and interfacial excess for each of these asymmetric (as well as symmetric) boundary structures shown in Fig. 5, where property values between these angles could be interpolated and compared to the experiments.

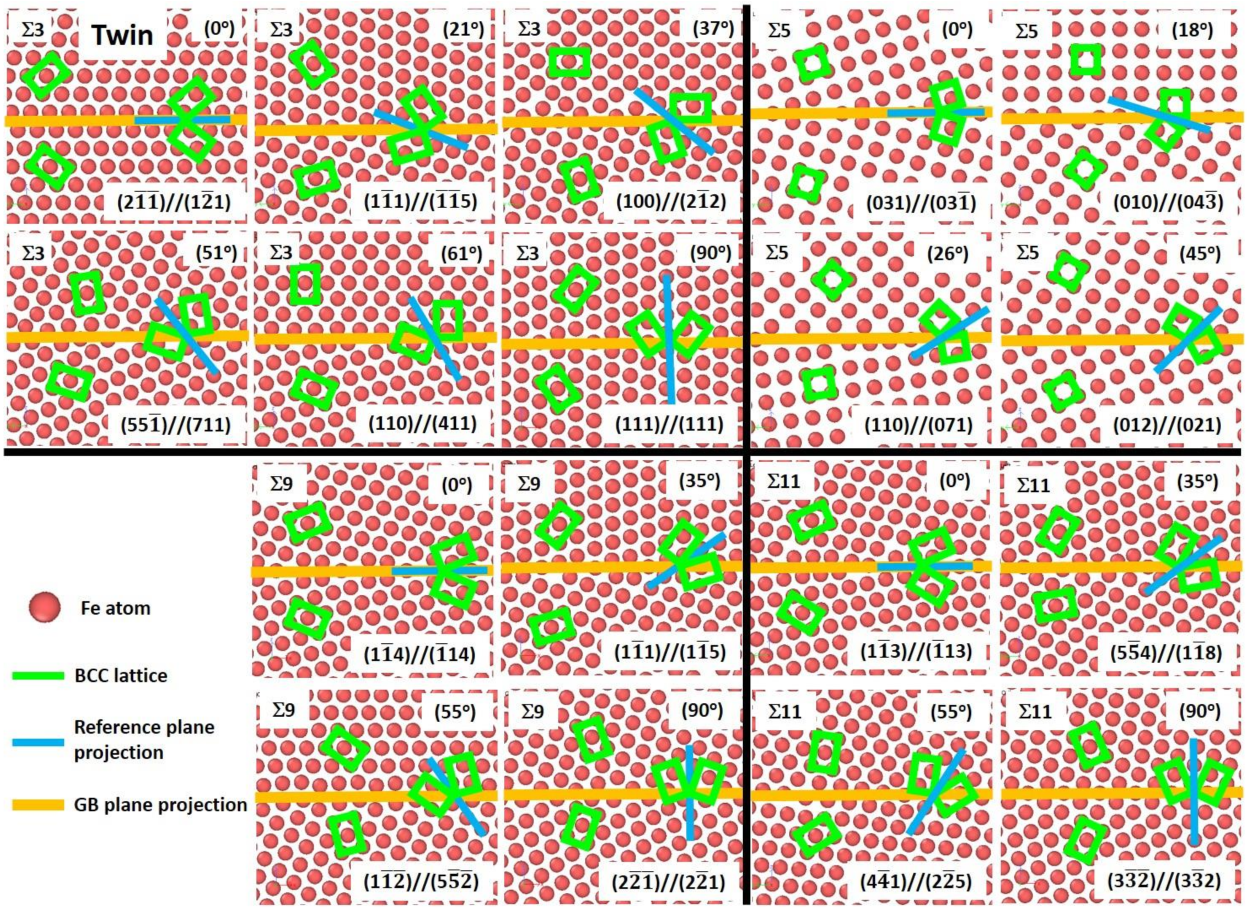


**Fig. S4:** Structural views of asymmetric Σ3, Σ5, Σ9 and Σ11 CSL grain boundaries. The green lines guides the eye for the lattice structure whereas the orange lines reveal the GB plane projection and the blue lines are the projected symmetric plane between the two unit cells between the two adjacent grains.

## 2.2 Reference inclination angle for experimental grain boundaries

To determine the grain boundary inclination angle from the experimental PED patterns, a reference plane must also be established between the two grains' orientations. To do this, the following procedure was performed. First, the grain boundary was defined by the grain boundary normal, which is represented by the red line in Fig. S5. This line can be assumed to be normal to the electron beam since the GB planes are reasonably parallel to the viewing direction, as

described above and shown in Fig. 4(a), or determined by the PED scan that provided the weighted diffraction patterns across the boundary that revealed the projection of the boundary [1]. The PED scan also provided the quantitative indices for each grain's texture relative to the beam's projection. This information was then exported and used to create the projection of the unit cell referenced to this texture using a crystallographic software package[2]. This is schematically shown in Fig. S5(a). Knowing the texture, the unit cells on either side of the CSL boundary are then rotated to a common viewing direction based on the CSL type as described above and is schematically shown in Fig. S5(b). After titling to this common viewing direction, the symmetric plane between the two unit cells is readily identified and can now serve as the reference plane needed to measure against the physical grain boundary as done for the computed asymmetric boundary types described above in 2.1. This is the projected blue line in Fig. S5(b). Since the grain boundary normal will rotate with the unit cell to the appropriate viewing direction, the GB plane is readily identified after rotation as the orange projected plane as schematically shown in Fig. S5(b). If the boundary was inclined (as measured by PED), then upon rotation of the unit cell to the common viewing direction, the grain boundary normal was adjusted accordingly to the inclination angle relative to the grain texture. For conditions were the GB was parallel to the beam (little to no inclination), this made the texture rotation, coupled with the grain boundary normal, straightforward because one does not need to be concerned with how much the boundary itself is inclined to the grain's texture orientation.  The experimental angle of inclination between the two grains that created the CSL type can then be measured between the reference (blue projected line trace) and the GB plane (projected orange surface) as shown in Fig. S5(b). The results of these findings are tabulated in Table 2 under the column 'Inclination angle (REF).'

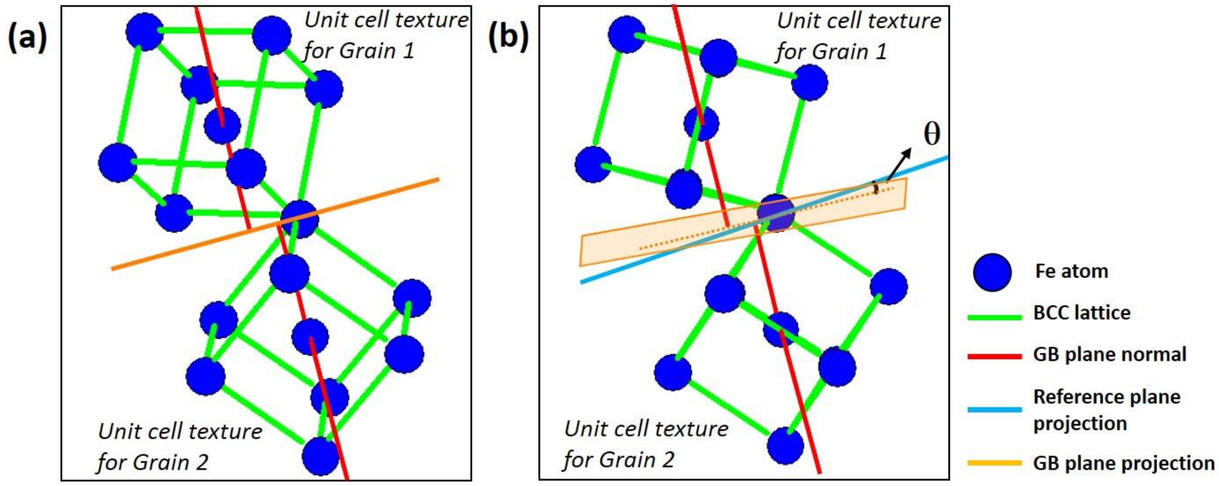


**Fig. S5: (a)** Schematic representation of the unit cell texture determined by the PED scan for a Σ3 twin. The red lines represent the normal to the grain boundary to each of the unit cell for each grain. **(b)** Schematic representation of the unit cell texture rotated to a common viewing direction from which the symmetric plane, represented by the projected blue line, can be found. The orange plane represents the projected grain boundary determined from the GB normal (red lines). The inclination angle, Θ, is measured from the projected symmetric plane (blue line trace) and the projected grain boundary plane (orange surface), represented by the dashed orange line.

3. Simulations result of additional CSL grain boundaries

Though these boundaries were not found in the experimental film, we have provided a series of additional simulations of GB energy, excess volume, and interfacial excess for other specific types of symmetric Σ-boundaries that are noted to occur in BCC Fe. This information is solely given to the reader as a larger data base or predicted Cr segregation in Fe based for those specific boundary types as shown in Fig. S6.

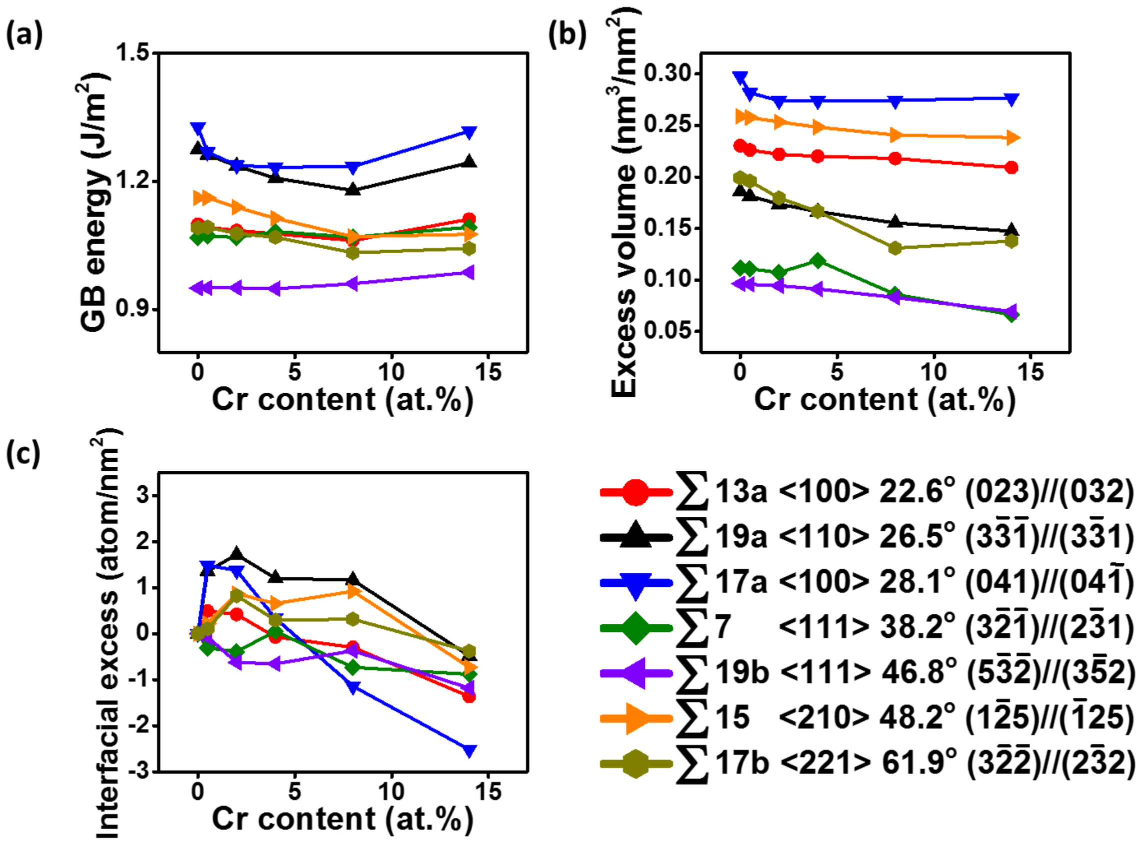


**Fig. S6: (a)** Calculated grain boundary energy **(b)** Excess volume **(c)** Interfacial excess for other Σ-GBs listed in the legend.

## References


1 Kiss, A. K. & Labar, J. L. Determining Projections of Grain Boundaries from Diffraction Data in Transmission Electron Microscope. *Microscopy and microanalysis : the official journal of Microscopy Society of America, Microbeam Analysis Society, Microscopical Society of Canada* **22**, 551-564, doi:10.1017/S1431927616000684 (2016).

2 Boudias, C. & Monceau, D. *CaRine Crystallography*, Compiègne: DIVERGENT SA. (1998).
